# Assessing recovery of spectacled eiders using a Bayesian decision analysis

**DOI:** 10.1371/journal.pone.0253895

**Published:** 2021-07-01

**Authors:** Kylee D. Dunham, Erik E. Osnas, Charles J. Frost, Julian B. Fischer, James B. Grand

**Affiliations:** 1 School of Forestry and Wildlife Sciences, Alabama Cooperative Fish and Wildlife Research Unit, Auburn University, Auburn, Alabama, United States of America; 2 U.S. Fish & Wildlife Service, Migratory Bird Management, Anchorage, Alaska, United States of America; 3 U.S. Geological Survey, Alabama Cooperative Fish and Wildlife Research Unit, Auburn University, Auburn, Alabama, United States of America; Office Français de la Biodiversité, FRANCE

## Abstract

Assessing species status and making classification decisions under the Endangered Species Act is a critical step towards effective species conservation. However, classification decisions are liable to two errors: i) failing to classify a species as threatened or endangered that should be classified (underprotection), or ii) classifying a species as threatened or endangered when it is not warranted (overprotection). Recent surveys indicate threatened spectacled eider populations are increasing in western Alaska, prompting the U.S. Fish and Wildlife Service to reconsider the federal listing status. There are multiple criteria set for assessing spectacled eider status, and here we focus on the abundance and decision analysis criteria. We estimated population metrics using state-space models for Alaskan breeding populations of spectacled eiders. We projected abundance over 50 years using posterior estimates of abundance and process variation to estimate the probability of quasi-extinction. The decision analysis maps the risk of quasi-extinction to the loss associated with making a misclassification error (i.e., underprotection) through a loss function. Our results indicate that the Yukon Kuskokwim Delta breeding population in western Alaska has met the recovery criteria but the Arctic Coastal Plain population in northern Alaska has not. The methods employed here provide an example of accounting for uncertainty and incorporating value judgements in such a way that the decision-makers may understand the risk of committing a misclassification error. Incorporating the abundance threshold and decision analysis in the reclassification criteria greatly increases the transparency and defensibility of the classification decision, a critical aspect for making effective decisions about species management and conservation.

## Introduction

The goal of the Endangered Species Act (ESA) [1] is to protect and recover imperiled species and the ecosystems upon which they depend so federal protection is not necessary for maintaining viability of the species. Recovery plans for species listed under the ESA are developed to provide guidance regarding management actions and must include objective measurable criteria to indicate when species reclassification (delisting or downlisting) is warranted. For many species, the measurable criteria for reclassification are based on abundance, trend, and extinction risk deemed appropriate by the species recovery team. Distinguishing when these criteria are met is inherent in the concept of setting measurable objectives and has significant implications for listed species and agencies tasked with their protection (e.g., U.S. Fish and Wildlife Services [USFWS], National Marine Fisheries Service [NMFS]).

Recent surveys indicated spectacled eiders (*Somateria fischeri*), listed as threatened under the ESA [2] have been increasing on one of their primary breeding grounds in Alaska, prompting the USFWS to consider population status relative to recovery criteria [3, 4]. The global population (i.e., the species) of spectacled eiders is listed and includes three distinct breeding populations in Arctic Russia, northern Alaska along the Arctic Coastal Plain (ACP), and western Alaska on the Yukon-Kuskokwim Delta (YKD) [5]. The species can be considered for delisting from threatened status following an analysis of continuous threats based on five factors ([1]; section 4(a)(1)(A-E)] and when each of the three breeding populations meets the quantitative criteria outlined in the species recovery plan [3, 4]. While the recovery plan suggests that the three breeding populations meet the distinct population segment (DPS) criteria [6], they were not formally designated as DPSs and thus, reclassification decisions must be made for the entire species [2]. Based on aerial or nest surveys the populations can be assessed by one of two sets of criteria: i) the minimum estimated breeding population size is ≥ 6,000 breeding pairs (or 12,000 breeding birds) designated by the 95% lower credible interval, and the overprotection loss exceeds the underprotection loss as determined by an analysis of trend data (10–15 years with 1 survey/year) and where loss functions are symmetrical around population growth *r* = 0 with zero loss for both functions when *r* = 0, or ii) the minimum population size is ≥ 10,000 breeding pairs over ≥ 3 surveys or the minimum estimate of abundance exceeds 25,000 breeding pairs in any survey (see Criteria for delisting from threatened status pp. 36–38 in [6]). Here, overprotection refers to the process of providing a species protection when it is not warranted, and underprotection refers to failing to provide protection when it is warranted [6]. Based on limited aerial surveys of the breeding and the wintering areas, the Russian breeding population is large (> 100,000 breeding birds) and estimates surpass the second criteria [7, 8]. By comparison, the two breeding populations in Alaska represent a smaller portion of the global population and their status relative to these criteria are unknown [3, 4]. However, since listing, the YKD breeding population has increased in abundance and may be close to meeting the first delisting criteria [3, 4, 6]. Determining if the Alaskan breeding populations have met the delisting criteria has wide-reaching implications for the species conservation status.

Species classification decisions are liable to two possible errors: i) failing to classify a species as threatened or endangered that should be classified (underprotection), or ii) classifying a species as threatened or endangered when it is not warranted (overprotection) [9]. Decision theory provides a framework for linking statistical inference on population metrics to the risk of making a classification error based on statistical results, expected consequences of the possible decisions (i.e., loss), and prior beliefs about the system [9–11]. The link between statistical inference and decision making occurs through the specification of a loss function that expresses the cost associated with the decision and the true state of nature [11, 12]. The spectacled eider classification problem consists of three alternatives or decisions; i) to delist the species, ii) maintain current (threatened) status, or iii) reclassify as endangered. Given the growth of the YKD population we consider alternatives one and two to determine the optimal decision based on the quantitative criteria. The analysis for considering reclassification from threatened to not warranted (i.e., delisting) is based on the specification of two loss functions which are symmetrical around a population growth of *r* = 0 (i.e., stable growth) as defined within the species recovery plan [6]. The symmetrical nature of these loss functions represents a choice made by the species recovery team to minimize potential bias and represents a risk neutral approach for evaluating outcomes. The first loss function represents the cost of underprotection and is calculated based on the risk of the population falling below a quasi-extinction threshold in 50 years. This function is grounded on the principle that species should be classified by the level of extinction risk. Extinction risk is nearly certain (1.0) when the population is declining rapidly and the risk of underprotection decreases as the risk of extinction decreases and reaches 0 when the population is stable or growing [6]. The second loss function represents the cost of overprotection and is simply the mirror image of the first loss function. Thus, loss associated with this second function increases as extinction risk decreases from 0, when the population is declining or stable, to its maximum (1.0), when there is no risk of extinction. The risk of committing a misclassification error (i.e., under or over-protection) is therefore calculated as the risk associated with each loss function integrated with the posterior distribution around the current population growth rate (*r*). For spectacled eiders, the listing decision is based on the comparison of overprotection risk and underprotection risk. Though alternative loss functions exist (see [9–12] for examples) the primary purpose of this study was to evaluate outcomes based on the established recovery criteria to inform status decisions.

Our work focused on determining if the Alaskan breeding populations of spectacled eiders have met the quantitative criteria outlined in the species recovery plan to consider delisting. Thus, we constructed alternative population models to estimate population metrics required to assess extinction risk, specifically, abundance, population growth rate, and process variation. Using these results, we conducted a decision analysis by calculating loss functions and misclassification error related to a decision to delist or maintain the species threatened status. We used alternative models to address concerns held by the USFWS about the effects of uncertainty in detection and observation processes on the decision analysis. The results from this study serve to inform classification decisions and conservation planning for Alaskan breeding spectacled eiders. Our approach combining population models with loss functions and accounting for uncertainty in observation processes is applicable to many species classification decisions that require not only quantitative assessments of population status but also value judgements and risk tolerance of decision makers.

## Materials and methods

We went through the following steps when conducting this analysis and describe each step in more detail in the sections below. First, we gathered detection adjusted abundance estimates and standard errors from aerial surveys of the ACP and YKD breeding populations of spectacled eiders from 2007 to 2019. We fit these data using Bayesian state-space models to estimate abundance, population growth rate, and process variation for both populations. We constructed 2 alternative models for the ACP breeding population and 4 alternative models for the YKD breeding population to reflect uncertainties in detection and observation processes. As a first step to evaluate if the recovery criteria were met, we determined if the lower 95% Bayesian credible interval (CRI) of abundance in 2019 was ≥ 12,000 breeding birds for each model. We then generated the loss function and calculated the probability of committing a misclassification error based on expected loss and the posterior of mean population growth rate. The results serve to provide managers with a robust and transparent assessment of spectacled eider status that may be used to inform species conservation decisions. All data and code used for this analysis are available in Table 1 and the S1 Appendix, respectively.

**Table 1:** Detection adjusted abundance estimates for Alaskan breeding populations of spectacled eiders (*Somateria fischeri*) from 2007 to 2019 provided as data ($\hat{y_{t}}$, the mean observed number of breeding birds and $\sigma_{\hat{y_{t}}}$, the estimated standard error for the number of breeding birds) in the observation model.

	YKD^a^		ACP^b^	
	*Number of breeding birds*		*Number of breeding birds*	
Year	$\hat{y_{t}}$	$\sigma_{\hat{y_{t}}}$	$\hat{y_{t}}$	$\sigma_{\hat{y_{t}}}$
2007	12,527	1,045	6,555	961
2008	14,580	1,273	7,733	939
2009	15,562	1,232	7,072	1,226
2010	13,491	1,056	6,892	987
2011	NA	NA	10,562	1,258
2012	14,696	1,279	6,228	679
2013	16,178	1,238	9,995	1,302
2014	13,152	1,075	9,651	1,382
2015	5,714	494	7,745	969
2016	14,481	1,086	5,696	892
2017	16,727	1,368	5,951	1,073
2018	15,544	1,241	6,418	1,276
2019	15,111	1,137	5,108	725

There were no surveys flown in 2011 and thus no estimates are provided.

^a^ YKD metrics refer to the Yukon-Kuskokwim Delta breeding population.

^b^ ACP metrics refer to the Arctic Coastal Plain breeding population.

### Survey methods

Aerial surveys have been flown annually since the early 1990’s to monitor both breeding populations of Alaskan spectacled eiders [3, 13, 14]. In most aerial surveys of waterfowl during the breeding season, waterfowl are recorded in such a way as to distinguish breeding birds from non-breeding birds. We used the number of indicated breeding birds which includes observations of single birds and pairs [3]. When aerial surveys are flown, spectacled eiders have typically been in pairs and in very few cases have flocks been documented on either breeding area. Following guidelines regarding the temporal scope for analysis in the recovery plan, and to include data from the most consistent sampling period across both study sites, we used survey data from 2007 to 2019 for both populations.

#### Arctic coastal plain breeding population surveys

The ACP spans approximately 90,000 km^2^ on the North Slope of Alaska bordering the Chukchi and Southern Beaufort Seas [13, 14]. USFWS Division of Migratory Bird Management conducts annual aerial surveys sampling nearly 60,000 km^2^ of the ACP to monitor the distribution, abundance, and trend of bird species. The ACP survey was flown annually following consistent methods from 2007 to 2019 [13, 14]. In 2015 and 2016, USFWS implemented double-observer techniques to estimate aerial detection probabilities of spectacled eiders breeding on the ACP (for methodological details, see [13]). We used detection-adjusted estimates of indicated breeding birds and error in our analysis (Table 1).

#### Yukon-Kuskokwim delta breeding population surveys

The YKD of western Alaska spans approximately 130,000 km^2^ and borders the Bering Sea [3, 4, 9]. Aerial surveys of spectacled eiders have been conducted over 12,832 km^2^ of YKD tundra wetland habitat annually since 1988 [3] Additionally, ground-based surveys have been conducted annually on the YKD since 1985 to estimate the numbers of nests for geese and eiders. This survey sampled randomly selected plots within the core nesting area of spectacled eiders in the central coast zone encompassing 716 km^2^ [3]. Estimates of nests and aerial observations among low, medium, and high-density stratum on the YKD were used to calculate density-specific aerial visibility correction factors (VCF) to account for incomplete detection on aerial surveys. Lewis et al. [4] converted the aerial indices of spectacled eider abundance to annual estimates of breeding abundance using the average density-specific visibility correction factors. The estimates generated for 2007–2019 were provided as observation data and error in our analysis (Table 1).

### State-space models

Our first goal was to estimate mean population growth rate, $\bar{r}$, and process or temporal variation in population growth rate *σ*_*r*_ using detection adjusted abundance estimates from aerial surveys of spectacled eiders on the YKD and ACP. We used Bayesian state-space models to partition population dynamics into two components, the hidden state process and the observation model, and fit the process model to the time series of observations [15, 16]. State-space models simultaneously account for both process variation and observation error caused by partial observability on surveys [15, 16].

The spectacled eider recovery team was interested in understanding the effects of model assumptions on population estimates and the decision analysis. Specifically, concerns were raised about the use and effects of informative and noninformative priors on the model estimates and decision analysis. Additionally, in 2015, a different observer conducted the eider aerial surveys on the YKD. The abundance estimate for 2015 indicated that the population dropped substantially from the previous year. However, upon closer inspection, the counts for that year were significantly smaller than previous and following years [4] and estimates from the nest counts for 2015 indicate no real decline in population size ([17], pg. 26 of report). Finally, the detection-corrected population estimates were based on the mean detection across years [4, 13]; thereby assuming that detection is constant. The observation of the 2015 data on the YKD as well as a large literature on population estimation (e.g., [18]), suggests that detection is rarely constant across years. Ignoring latent observation processes has been shown to bias estimates of demographic parameters [19]. Given these concerns, we fit a total of 6 models: 2 for the ACP population and 4 for the YKD population. Models ACP1 and YKD1 included all available years of data between 2007 and 2019 and were initialized with ‘informative’ priors based on the species’ biology and expert opinion (Table 2). Model YKD2 included all years of data between 2007 and 2019 and was initialized using noninformative priors (Table 2). Model YKD3 was fit by excluding the 2015 population estimate and initializing the model with informative priors. Finally, models ACP2 and YKD4 allowed for a latent observation process and used informative priors to provide estimates of VCF variance and an observer effect (Table 2). Model parameters and prior distributions are described in Table 2. We worked closely with the eider recovery team throughout all stages of the analysis including, but not limited to considerable discussion regarding the choice of priors and alternative models. Subsequently, informative priors were based on species biology and informally elicited expert opinion from the recovery team.

**Table 2:** Model descriptions, parameters, and prior distributions used to model population dynamics of spectacled eiders (*Somateria fischeri*) breeding on the Arctic Coastal Plain (ACP) and Yukon-Kuskokwim Delta (YKD).

Model	Model Description	Parameters	Prior Distributions
ACP1	Informative priors based on expert opinion and species biology	log(*N*_2007_)	*Normal* (8.78, 0.1)
		$\bar{r}$	*Normal* (0, 0.1)
		$\sigma_{r}^{2}$	*Gamma* (3, 20)
ACP2	Informative priors (see above) and includes latent variation in the observation process	log(*N*_2007_)	*Normal* (8.78, 0.1)
		$\bar{r}$	*Normal* (0, 0.1)
		$\sigma_{r}^{2}$	*Gamma* (3, 20)
		*σ*_*d*_	*Gamma* (1, 10)
YKD1	Informative priors based on expert opinion and species biology	log(*N*_2007_)	*Normal* (9.43, 0.1)
		*r*_*t*_	*Normal* (0, 0.1)
		$\sigma_{r}^{2}$	*Gamma* (3, 20)
YKD2	Noninformative (diffuse) priors	log(*N*_2007_)	*Normal* (9.43, 0.5)
		$\bar{r}$	*Normal* (0, 0.5)
		$\sigma_{r}^{2}$	*Gamma* (3, 2)
YKD3	Informative priors with 2015 observation removed	log(*N*_2007_)	*Normal* (9.43, 0.1)
		$\bar{r}$	*Normal* (0, 0.1)
		$\sigma_{r}^{2}$	*Gamma* (3, 20)
YKD4	Informative priors and includes latent variation in the observation process in the form of random effects and includes a fixed effect for the new observer in 2015	log(*N*_2007_)	*Normal* (9.43, 0.1)
		$\bar{r}$	*Normal* (0, 0.1)
		$\sigma_{r}^{2}$	*Gamma* (3, 20)
		*σ*_*d*_	*Gamma* (1, 10)
		*Β*	*Gamma* (15.5, 0)

Here, we describe each model, the relevant parameters, and the respective prior distributions. The parameters include, *N*_2007_– population size in 2007, $\bar{r}$ – mean population growth rate, $\sigma_{r}^{2}$ – process variance, $\sigma_{d}^{2}$ – standard deviation of annual VCF, and *β* –the fixed effect of a new observer. For the *Normal* distributions we report the mean and the standard deviation on the log scale. The *Gamma* distributions are reported with the shape and rate parameters. For the process variance parameter ($\sigma_{r}^{2}$) we report the prior distributions for the standard deviation.

We modeled the log initial abundance as the log of the point estimate for abundance in 2007 the first year of our time series, with a standard Normal prior either 0.1 or 0.5 to generate an informative or noninformative distribution, respectively. The prior for mean population growth rate (*r*) is Normal with mean 0 with standard deviation is 0.1 or 0.5 for models with informative or noninformative priors, respectively (S1 Fig in S2 Appendix). The standard deviation for informative priors for initial abundance and population growth rate were based on the initial abundance estimate and input from species experts. The prior distribution for temporal variation in population growth (here, process variance) was Gamma distributed and based on shape and rate parameters with a range of values between approximately 0.005 and 1 for models using informative priors and a range of values between 0.005 and 10 for models using noninformative priors (S2 Fig in S2 Appendix). Comparisons of the informative and noninformative priors with their respective models’ posterior distributions for *r* and process variance are included in the supplementary material (S2 Appendix) for transparency (S3 and S4 Figs in S2 Appendix). Descriptions of the priors for latent observation processes are described in further detail in the text below.

Each of the six state-space models described population growth as

$$\log\left(N_{t+1}\right)=\log\left(N_{t}\right)+r_{t}$$ (1)

where *N*_*t*_ is the number of breeding birds in year *t*, *r*_*t*_ is population growth rate, and

$$r_{t}∼Normal(\bar{r},\sigma_{r}^{2}).$$ (2)

The observation model relates the true population size *N*_*t*_ to the observations corresponding to the detection-adjusted abundance indices for each breeding area. For models ACP1 and YKD1, YKD2, and YKD3, our observation process was

$$\hat{y_{t}}∼Normal(N_{t},{\hat{\sigma }}_{\hat{y_{t}}})$$ (3)

where the observations, $\hat{y_{t}}$, were the detection-adjusted abundance point estimates of spectacled eiders from the aerial surveys on the respective breeding grounds (i.e., ACP and YKD) [3, 14] (Table 1). Annual observation error from aerial survey sampling (${\hat{\sigma }}_{\hat{y_{t}}}$) was provided as data (see similar approach in [20]) (Table 1).

An alternative observation model was required to account for the latent observation processes for models ACP2 and YKD 4. For the ACP, we simply added a multiplicative random effect (*d*_*t*_) to population size (*N*_*t*_) and the associated prior for the variance of this effect (*σ*_*d*_) in the observation model

$$\hat{y_{t}}∼Normal(\frac{N_{t}}{d_{t}},{\hat{\sigma }}_{{\hat{y}}_{t}})$$ (4)

$$\log(d_{t})∼Normal(0,\sigma_{d})$$ (5)

$$\sigma_{d}∼Gamma(1,10).$$ (6)

In this model, *d*_*t*_ is the (unmeasured) year-specific deviation in the VCF. Positive deviations mean that fewer birds were observed ($\hat{y_{t}}$) relative to the population (*N*_*t*_). For the YKD, we modeled the observation process with the same random effect but added a fixed effect for the new observer in 2015

$$\log\left(d_{t}\right)∼Normal(\beta x_{t},\sigma_{d})$$ (7)

where *x*_*t*_ is an indicator equal to zero in all years except 2015, when it is 1, and *β* is a fixed effect regression parameter for the effect on VCF in 2015 due to a new observer. We used a prior for β informed by our knowledge of the number of eider nests estimated from ground-based surveys in 2015 [17].


β−1∼Gamma(15.5,9) (8)

We derived the parameters for this distribution by matching the mean and standard deviation based on the ratio of twice the estimated nests reported for 2015 (e.g., the expected number of breeding birds, 15,584 ± 2,472, [17]) to the estimate of indicated breeding birds derived from the VCF-corrected aerial data for 2015 (5,714 ± 494) which results in a ratio with mean 2.73 and standard deviation 0.49. With this information, we generated a Gamma prior for *β*−1 with the shape parameter 15.5 and rate parameter 9 which has a mean of 1.72 and standard deviation of 0.44 to limit the range of expected values from this ratio on the log scale.

The prior for *σ*_*d*_ in both the ACP and YKD models was chosen to reflect a belief that annual deviations in detection are most likely small but could be large with low probability. Combining Eqs (5) and (6) results in a prior distribution for the VCF deviations, *d*_*t*_, where 50% of |*d*_*t*_| < 0.04 and 99% are < 0.44. This is an extremely informative prior and implies most deviations are small but large deviations (> 0.04) can occur and very large deviations (>0.44) very rarely occur. In the absence of direct measures of annual VCFs, we believe this is a reasonable prior.

We fit the state-space models in a Bayesian framework implementing Markov chain Monte Carlo methods (MCMC, [21]) to sample the posterior distributions in *JAGS 3*.*3*.*0* ([22], using the *jagsUI* package in R [23]). We ran three MCMC chains for 100,000 iterations, set thin to 2, discarded 70,000 iterations as burn-in, and ran 5,000 iterations in the JAGs adaptive phase. We checked convergence using the Gelman-Rubin statistic [24] and all results were satisfactory (all $\hat{R}$ <1.01).

### Decision analysis

The basic elements in statistical decision analyses include *θ*, the state of nature which affects the decision process, and Θ is the set of all possible states of nature [11, 12]. The decisions or actions are denoted by *a*, and all possible actions considered may be denoted A. The loss function, *L*(*θ*, *a*) describes the loss associated with taking action *a* for each *θ* state of nature and the function is defined for (θ,a)∈Θ*A. Following Berger [12] and Williams and Hooten [11], the general notation for Bayesian expected loss is:

$$E_{\theta |y}L(\theta ,a)=\int_{\Theta }L(\theta ,a)[\theta |y]\;d\theta .$$ (9)

In this analysis we consider two possible states of nature based on the recovery criteria, the first is that the population is declining (*r*<0) and the second is that the population is stable or increasing (*r*≥0). Additionally, there are two alternative actions (*a*) which refer to delisting or maintaining the threatened status. The recovery team chose symmetric loss functions around *r* = 0 to represent equivalent loss associated with providing too little (underprotection) or too much (overprotection) protection to the species based on the classification of threatened or not warranted [6, 9]. The loss functions refer to the decision to delist when the population is declining (i.e., underprotection) and for the decision to maintain the threatened status when the population is stable or increasing (i.e., overprotection). The loss associated with delisting spectacled eiders is equivalent to the probability of reaching a quasi-extinction threshold of 250 breeding birds within 50 years, given a projection using the abundance and process variation estimates from the state space model(s) over a range of population growth rates with a zero-loss occurring once *r* = 0. Loss is set to zero once *r* = 0 because the recovery team decided that the decision to delist is correct when the population is stable or increasing. Based on the decision to equalize errors we set the loss function for overprotection to reflect the underprotection loss function as specified by the USFWS [6] and described in Taylor et al [9]. The overprotection loss function is thus the loss incurred when the decision is to maintain the threatened status and the population is stable or increasing and there is zero loss when the population is declining because the decision to maintain the status is correct. In classification decisions, expected loss (e.g., under or over-protection loss) is also known as the conditional risk of committing a classification error. For this classification decision, expected loss or risk of committing a classification error is conditional on the posterior distribution of mean population growth rate generated from the state-space model(s).

We generated the underprotection loss function by projecting abundance for 50 years over all possible values of population growth denoted *rs* (all possible values of the state of nature, Θ). The range of *rs* was set from -0.4 to 0.4 because the posterior distribution of mean *r* produced from the state-space models fell within these limits. First, initial abundance is chosen from the posterior distribution of *N*_2019_ (see Table 3 for values); (ii) process variance *σ*_*r*_ is selected from the posterior distribution generated in the state-space model; (iii) *r*′ is pulled from *N*(*rs*, *σ*_*r*_) for each year; (iv) the population is projected forward for 50 years, when N falls below 250 breeding birds the run is assigned a 1 and if it does not reach this threshold within 50 years the run is assigned a 0; and (v) steps i-iv are repeated 10,000 times, and the number of times N falls below the quasi-extinction threshold is saved. The probability of committing a classification error is calculated as the sum of the expected loss for the decision to delist (underprotection loss) for *r*<0 and for the decision to maintain status (overprotection loss) for *r*≥0 multiplied by the probability of that value of *r* from the posterior distribution on mean population growth. This results in a single value (i.e., loss or the risk of committing a classification error) for each decision (i.e., to delist or maintain threatened status). Based on the specification of our loss functions and decision criteria, the optimal decision minimizes underprotection loss in favor of maximizing overprotection loss.

**Table 3:** Posterior estimates of population metrics and misclassification error for both Alaskan breeding populations of spectacled eiders (*Somateria fischeri)*.

	ACP1^a^	ACP2^a^	YKD1^b^	YKD2^b^	YKD3^b^	YKD4 ^b^
Abundance						
*Posterior mean*	5355	6401	15054	15047	15388	16113
*Posterior SD*	629	1510	1104	1118	908	2249
*95% CRI*	4106–6589	3766–9750	12903–17212	12863–17253	13595–17175	12313–21352
Population growth rate ‘r’						
*Posterior mean*	-0.016	-0.005	0.009	0.013	0.013	0.016
*Posterior SD*	0.043	0.043	0.068	0.137	0.023	0.037
*95% CRI*	-0.103–0.072	-0.092–0.082	-0.124–0.0143	-0.263–0.287	-0.035–0.062	-0.065–0.091
Process variation						
*Posterior mean*	0.158	0.142	0.323	0.479	0.073	0.123
*Posterior SD*	0.061	0.064	0.064	0.137	0.038	0.073
*95% CRI*	0.057–0.293	0.039–0.288	0.219–0.468	0.284–0.814	0.017–0.161	0.026–0.305
Loss						
*Underprotection*	0.181	0.108	0.145	0.282	0.0002	0.011
*Overprotection*	0.072	0.088	0.218	0.357	0.0061	0.068

Here, we report the mean (posterior mean), standard deviation (posterior SD), and 95% Bayesian credible intervals (95% CRI) for each parameter and model. Consideration for reclassification from threatened to recovered requires that both populations must reach or exceed the abundance threshold (N ≥ 12,000 breeding birds), and overprotection loss must be greater than underprotection loss. Abundance estimates and misclassification error rates for the ACP population do not support the decision to delist. Alternatively, all four models for the YKD population support delisting based on population metrics meeting the reclassification criteria in the species recovery plan.

^a^ ACP metrics refer to the Arctic Coastal Plain breeding population.

^b^ YKD metrics refer to the Yukon-Kuskokwim Delta breeding population.

## Results

**Fig 1:**
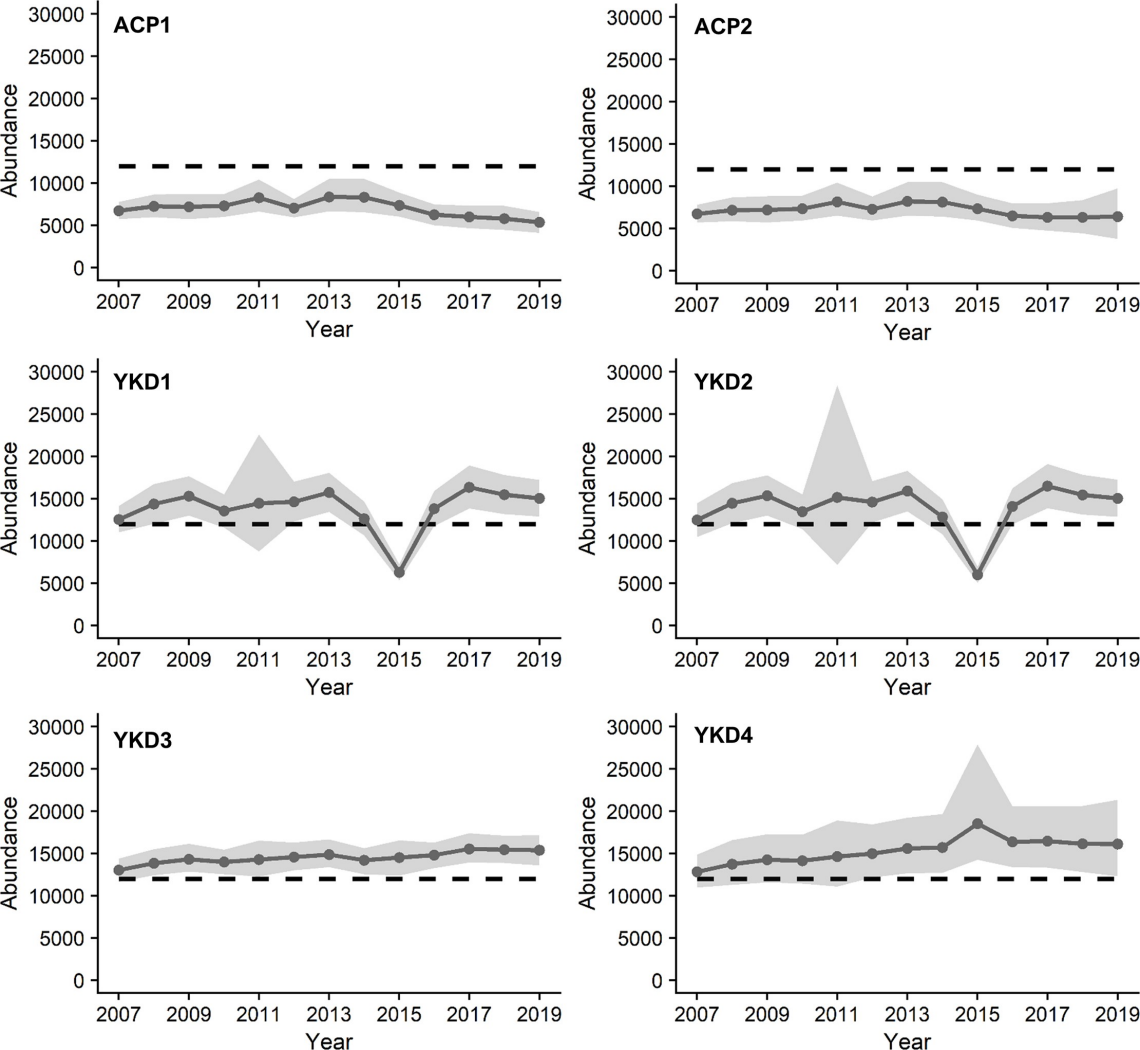
Posterior estimates of abundance for spectacled eider populations breeding on the Arctic Coastal Plain (ACP) and Yukon-Kuskokwim Delta (YKD) of Alaska. We fit 2 alternative models for the ACP breeding population and four alternative models for the YKD breeding population. Models ACP1 and YKD1 included all available years of data between 2007 and 2019 and were initialized with ‘informative’ priors based on the species’ biology and expert opinion. Model YKD2 included all years of data between 2007 and 2019 and was initialized using noninformative priors. Model YKD3 was fit by excluding the 2015 population estimate and initializing the model with informative priors. Finally, models ACP2 and YKD4 allowed for a latent observation process and used informative priors to provide estimates of VCF variance and an observer effect. Gray circles represent the annual mean abundance and gray ribbons represent the 95% credible interval (CRI). The black dashed line is the population threshold of 12,000 breeding birds identified in the species recovery plan.

**Fig 2:**
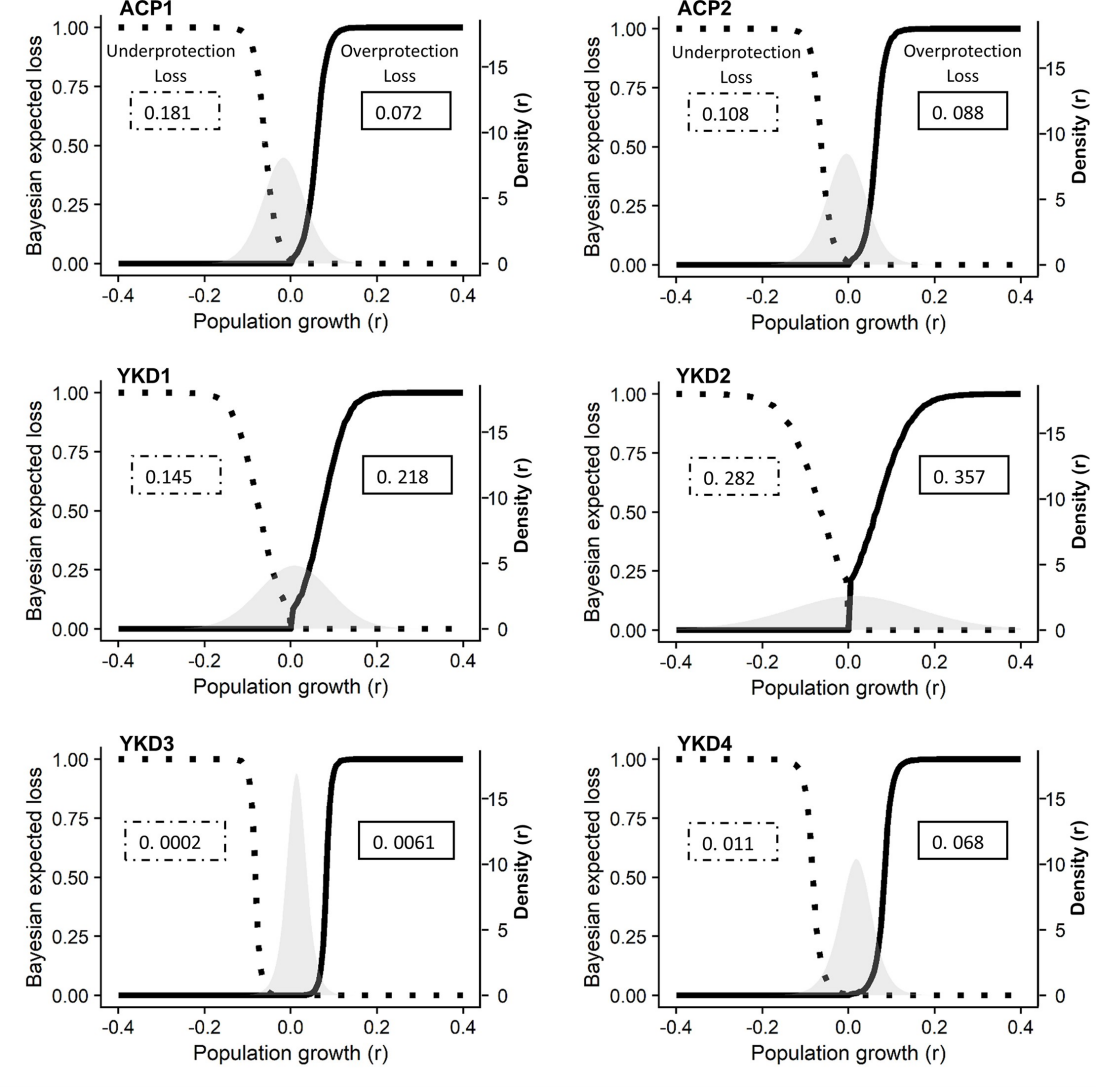
Loss functions and posterior distributions of population growth rate (r) generated from state-space models of abundance for spectacled eiders breeding on the Arctic Coastal Plain (ACP) and Yukon Kuskokwim Delta (YKD). Loss functions were generated using the probability of quasi-extinction given population size, growth rate, and process variation. The dotted line represents the under-protection loss function (i.e., loss if decision were to delist given negative population growth) and the solid line is the over protection loss function (i.e., loss if the decision were to maintain status given positive population growth). Gray distributions show the posterior density of population growth rate (*r*) estimated by a Bayesian state-space model for the time series from 2007 to 2019. As part of the recovery criteria, spectacled eiders will be considered for delisting if overprotection (value in solid line box) is greater than underprotection (value in dashed line box). Greater overprotection error indicates that we are more likely to provide too much protection to the species than we are to provide too little protection to the species.

Our results indicate that estimates of abundance and misclassification error are sensitive to uncertainty, but conclusions broadly remain consistent. Specifically, in each case the YKD population met the recovery criterion, whereas the smaller ACP population did not meet either the abundance threshold or the requirements for misclassification error (Table 3, Figs 1 and 2). The low abundance estimate combined with a highly variable and slightly decreasing population growth rate at the posterior mean for the ACP breeding population increase the risk of quasi-extinction. For the ACP breeding population, an underprotection error is more likely if the decision is to delist than an overprotection error if the decision is to maintain threatened status. The YKD breeding population is larger than the ACP population, and for each alternative model the lower 95% CRI of the 2019 abundance estimate met the threshold of 12,000 breeding birds. However, similar to the ACP population results, the posterior of mean population growth rate for the YKD was uncertain and centered nearly at zero for each model (Table 3). While overprotection loss for the YKD population is larger than underprotection loss for each alternative model, there is still considerable uncertainty in population metrics when the low estimate from 2015 is included in the data and detection is assumed constant (Models YKD1, YKD2).

### Arctic coastal plain population

Analysis of the ACP data indicates the population has not met any of the recovery criteria (Table 3). When assuming constant detection, estimated posterior mean abundance for the ACP population in 2019 was 5,355 breeding birds (95% CRI 4,106–6,589; Model ACP1, Table 3, S5 Fig in S2 Appendix). When we allowed detection to vary across years, estimated mean abundance in 2019 was 6,401 (3,766–9,750; Model ACP2, Table 3, S5 Fig in S2 Appendix). Based on estimated abundance in 2019 for either model, the ACP population has not met the abundance threshold ($95\%\;\mathrm{CRI}=\hat{N}$ ≥ 12,000 breeding birds) (Fig 1). The posterior mean for mean growth rate of the ACP population was negative ($\bar{r}=‐0.016$ or -0.005) with wide credible intervals (95% CRI: -0.103 to 0.072; -0.092 to 0.082) for models assuming constant and variable detection, respectively (Table 3). Furthermore, underprotection loss for the ACP population was greater than overprotection loss in both models (Table 3, Fig 2).

### Yukon-Kuskokwim delta population

We fit four alternative models to the YKD breeding population data. In each case, estimates of abundance exceeded the decision threshold and overprotection loss exceeded underprotection loss, indicating the population has met the recovery criteria. Model YKD1 includes all data points between 2007 and 2019 and was initialized with informative priors (see State-space models section in Materials and methods). Estimated posterior mean abundance from model YKD1 in 2019 was 15,054 breeding birds (95% CRI 12,903 to 17,212) (Table 3). The 95% lower CRI of abundance in 2019 is above 12,000 breeding birds indicating the YKD breeding population met the abundance threshold criterion (Fig 1 and S6 Fig in S2 Appendix). The posterior mean for mean population growth rate of the YKD population was $\bar{r}$ = 0.009 with a wide credible interval (95% CRI -0.124 to 0.143) (Table 3, Fig 2). Overprotection loss was 1.5 times that of underprotection loss based on the YKD1 model (Table 3, Fig 2).

Model YKD2 included all data points between 2007 to 2019 and was initialized with noninformative priors. Estimated posterior mean abundance for 2019 from model YKD2 was 15,047 (95% CRI 12,863 to 17,253), which exceeds the abundance threshold (Table 3, Fig 1 and S6 Fig in S2 Appendix). The posterior mean of mean population growth rate was $\bar{r}$ = 0.013 and 95% CRI of -0.263 to 0.287. The posterior mean for process variation in this model was 0.479 (95% CRI of 0.280 to 0.801). Both the posterior mean growth rate and process variance are larger than those produced when using biologically realistic informative priors (Fig 2). Overprotection loss again exceeded underprotection loss and the risk of committing an overprotection error was 1.26 times that of the risk of committing an underprotection error.

Model YKD3 was initialized with informative priors and the 2015 data point was removed from the time series and treated as a missing data point similar to 2011 when no survey was conducted. The abundance estimate for 2019 from model YKD3 was also above the threshold, with a posterior mean of 15,388 and 95% CRI of 13,595 to 17,175 (Table 3, Fig 1 and S6 Fig in S2 Appendix). The posterior of mean population growth rate was substantially more precise than posterior distributions produced by YKD1 and YKD 2. The posterior mean of mean population growth was 0.013 with 95% CRI of -0.035 to 0.062 (Fig 2). The posterior mean process variation was only 0.073 (95% CRI of 0.017 to 0.161), significantly lower than estimates from YKD1 and YKD2 (Table 3). Overprotection loss exceeded underprotection loss, however, in this case the risk of committing an overprotection error was 30.5 times that of the risk of committing an underprotection error for model YKD3.

Finally, Model YKD4 included all data, informative priors for all parameters and latent variation in VCF. The posterior mean abundance for YKD4 in 2019 was 16,113 (95% CRI of 12,313 to 21,352) just satisfying the abundance threshold and substantially wider than the other models (Table 3, Fig 1 and S6 Fig in S2 Appendix). The posterior mean of mean population growth rate was 0.016 and 95% CRI of -0.065 to 0.091. For process variation, the posterior mean for YKD4 was 0.123 and 95% CRI of 0.026 to 0.305. Overprotection loss exceeded underprotection loss and the risk of committing an overprotection error given model YKD4 was 6.18 times that of the risk of committing an underprotection error.

## Discussion

We constructed a series of models to assess the status of two breeding populations of spectacled eiders in Alaska and determine if the populations had met the species recovery goals. Our results demonstrated that the ACP population of spectacled eiders has not met the quantitative criteria required to consider delisting; however, the YKD breeding population has met the recovery criteria. Our application of a decision analysis in conjunction with a population assessment is an example of a robust methodology for informing species classification decisions based on population estimates in addition to value judgements and risk tolerance. Furthermore, our approach including alternative models offered an opportunity to explore the effects of uncertainty not only on population estimates but also on the risks associated with species classification decisions.

When using population metrics (abundance, trends, demographic rates, etc.) for species classifications, harvest regulations, or other management actions, it is important to consider the accuracy and precision of those estimates and the influence those estimates may have on a decision [25]. A considerable portion of variation in the YKD population growth rates and process variation can be attributed to the negative bias introduced by the 2015 data and assuming constant VCFs across years (Tables 1 and 2, Fig 2). Beginning in 2015, a new observer was assigned to conduct the aerial surveys and VCF values tended to be elevated in years with new observers which in conjunction with low counts biased the abundance estimate low [4]. Furthermore, the VCF accounted for nesting density and spatial variation but did not account for temporal variation in detection or a fundamental change in study design (i.e., a new observer). Removing the biased abundance estimate from 2015 (model YKD3) or accounting for temporal variation in detection (model YKD4) had a considerable effect on the precision of population metrics and on the risk of committing an overprotection error. There is considerable information to suggest that the perceived decline in 2015 was the result of an unaccounted-for change in observation process and not a true decline in population size or growth rate. In addition to the abnormally low counts and the evidence of unmodeled variation in observation processes from this analysis, estimates from the YKD nest plot survey estimated greater than 7,000 nests (14,000 breeding birds) in 2015 ([17], pg. 26 of report). This analysis highlights two important points: (i) in models where we use a constant VCF and do not account for temporal variation or observer changes, residual variation in the data is captured by the process variation term [26, 27] and it is biased high related to the year-specific variance in the VCF; therefore, (ii) the extinction risk, loss functions, and risks of committing a misclassification error reported here are also biased high. By extending the analysis to consider multiple models and assumptions, we explicitly incorporated the effects of uncertainty into the decision analysis and population assessment and provide decision makers with transparent results. Importantly, we note that the results consistently showed that the ACP population did not meet the recovery criteria and the YKD population did meet the recovery criteria, regardless of the underlying model assumptions.

By fitting models ACP2 and YKD4 that included year-specific variation in detection (VCF) as a random effect (both models) and fixed effects for systemic changes in study design (e.g., novice observers; model YKD4) we were able to account for latent variation in observation processes. In the context of N-mixture models, Zhao and Royle [19] found that assuming constant detection when in fact detection varied annually caused biased estimates of demographic parameters. They also found that fitting a model with latent random effects for detection even with only one survey replicate per year reduced bias in demographic estimates. This is consistent with our state-space models using the eider data and latent effects for detection where we found that when a greater proportion of variation is attributed to the latent observation process, it results in a lower estimate of process variance, more precise estimates of mean growth rate, and reduced precision of the population estimates (Models ACP2 and YKD4, Table 3, S5 and S6 Figs in S2 Appendix). The results of Zhao and Royle [19] and our explorations suggest that not accounting for such variation in detection, either by direct measurement or by modelling the effect through a latent process, may cause abundance estimates to be too precise and our demographic parameters to be biased; thus, biasing population viability analyses. The former leads to overconfidence in the population estimate and incorrect decisions based on abundance thresholds, and together, both lead to biased estimates of extinction risk. While further research and consideration of how to treat this type of data when year-specific detection is not measured may be warranted, using informed priors based on expert judgement or auxiliary data for unmeasured processes seems a reasonable approach to improve conservation decisions. In any case, measurement of year-specific detection probability would increase the ability to appropriately account for uncertainty in both the observation and population process; thereby, leading to less biased population parameters and better management decisions.

We followed the decision analysis approach outlined in the spectacled eider recovery plan and in Taylor et al. [9] to quantitatively assess spectacled eider populations against recovery criteria. These criteria include the loss associated with a listing decision and an abundance threshold based on the lower 95% credible interval (i.e., 2.5 percentile) of the posterior distribution of abundance. Certain properties of the current recovery criteria, however, might be reevaluated as the recovery plan is revised to ensure that they reflect the current risk values of the decision makers. First, using loss has been proposed for endangered species listing decisions and other natural resource management problems, but has not yet been widely adopted [10, 11, 28]. We agree with this approach as it offers a transparent and logically coherent framework for making species classification decisions [11, 12]. However, we wonder if the choice of symmetrical loss functions around zero mean population growth with zero loss at *r* = 0 and a shape determined by a population viability analysis directly reflect the risk values of the decision makers. Loss functions can take many shapes that represent risk attitudes but can be difficult to elicit [11], and we suggest that the current loss functions might be improved or at least reevaluated. Second, setting loss equal to zero when *r* = 0 does not accurately reflect the extinction risk of the population which predicates the underprotection loss function. Specifically, when *r*≥0 there is still a non-zero risk of the population declining below the quasi-extinction threshold within the 50-year period and this risk is not accounted for in the current calculation of loss. Third, the abundance threshold criteria seem redundant to a criterion based on population viability because viability is based on growth rate, stochasticity in growth rate (here process variance), and current abundance. Importantly, the abundance threshold criteria is sensitive to the posterior variance in abundance, and in the absence of year-specific detection estimates, we may be greatly underestimating this variance. Furthermore, choosing an abundance threshold implies a loss function for abundance. The current 2.5 percentile threshold (the lower bound of a 95 percent credible interval) implies that overestimates are 39 times worse than underestimates of abundance under a linear loss function (see Table 2 of [11]). While it is certainly reasonable that overestimating a listed species’ abundance is worse than underestimating, using the same threshold as is widely used for statistical hypothesis testing, which is often devoid of any applied decision context, might be reconsidered. Even though the abundance threshold might seem simple and value-free, there is an implied value judgment for risk tolerance of the decision maker. If the variance in the abundance estimate is much larger than previously thought, it could influence decisions regarding status.

Decision-makers are often tasked with choosing conservation or management actions despite uncertainty. The methods employed here provide an example of accounting for uncertainty in such a way that incorporates both science and value-based judgements to inform decision-makers about the risk of committing a misclassification error [28]. Accounting for the uncertainty in population dynamics and observation processes in the population assessment and decision analysis allowed us to explore the impacts of those uncertainties in a robust and transparent manner. The combined strengths in these approaches provide a robust framework for formally linking ecological inference to conservation and management decisions under considerable uncertainty [9–11, 29]. We believe our approach is a reasonable method for capturing risk of listing decision alternatives with careful thought and explicit definitions of the loss functions and risk tolerance. Future applications may consider explicitly modeling the effects of different decisions on future population outcomes and incorporate these predictions with loss functions thereby representing decision makers’ risk tolerances to better inform listing status decisions. This analysis adds to the growing support for decision-theoretic approaches in applied ecology and conservation, and further emphasizes the importance of exploring the effects of uncertainty on making endangered species classification decisions.

## Supporting information

S1 Appendix Spectacled eider decision analysis R and Jags code. (RMD)

S2 Appendix Spectacled eider decision analysis supplementary figures. (DOCX)
