# Supplementary material for: Assessing recovery of spectacled eiders using a Bayesian decision analysis
Source: PLoS One. 2021 Jul 1;16(7):e0253895. doi: 10.1371/journal.pone.0253895 (PMC8248636; doi:10.1371/journal.pone.0253895)
Supplement: S2 Appendix — (DOCX) [file pone.0253895.s002.docx]

Assessing recovery of spectacled eiders using a Bayesian decision analysis

Kylee D. Dunham, Erik E. Osnas, Charles Frost, Julian B. Fischer, and James B. Grand

**Supporting information**

**S2. Appendix. Spectacled eider decision analysis supplementary figures.**


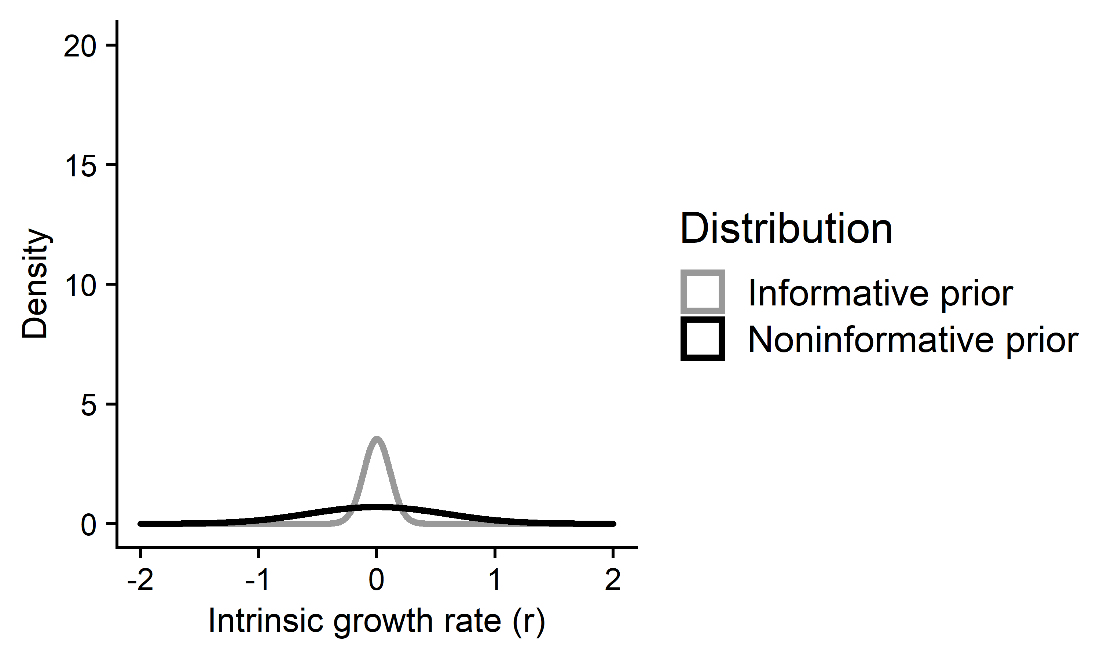


Fig S1. Prior distributions for population growth rate (*r*) used to initialize models of population dynamics for spectacled eiders on the Arctic Coastal Plain and Yukon Kuskokwim Delta.


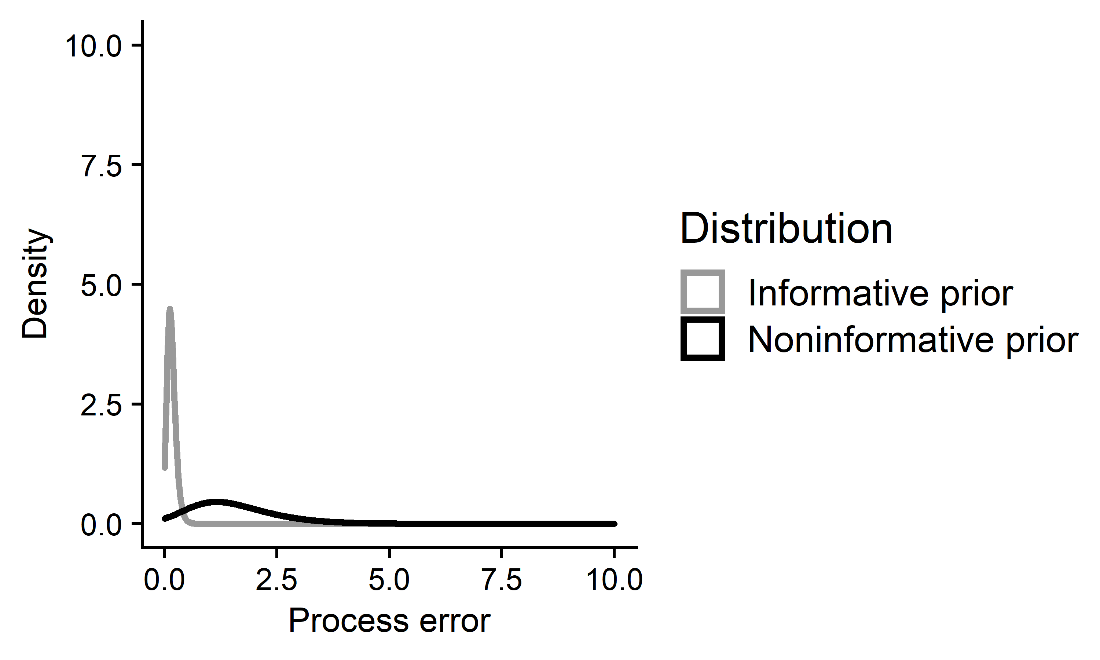


Fig S2. Prior distributions for process variance (e.g., process error) used to initialize models of population dynamics for spectacled eiders on the Arctic Coastal Plain and Yukon Kuskokwim Delta.


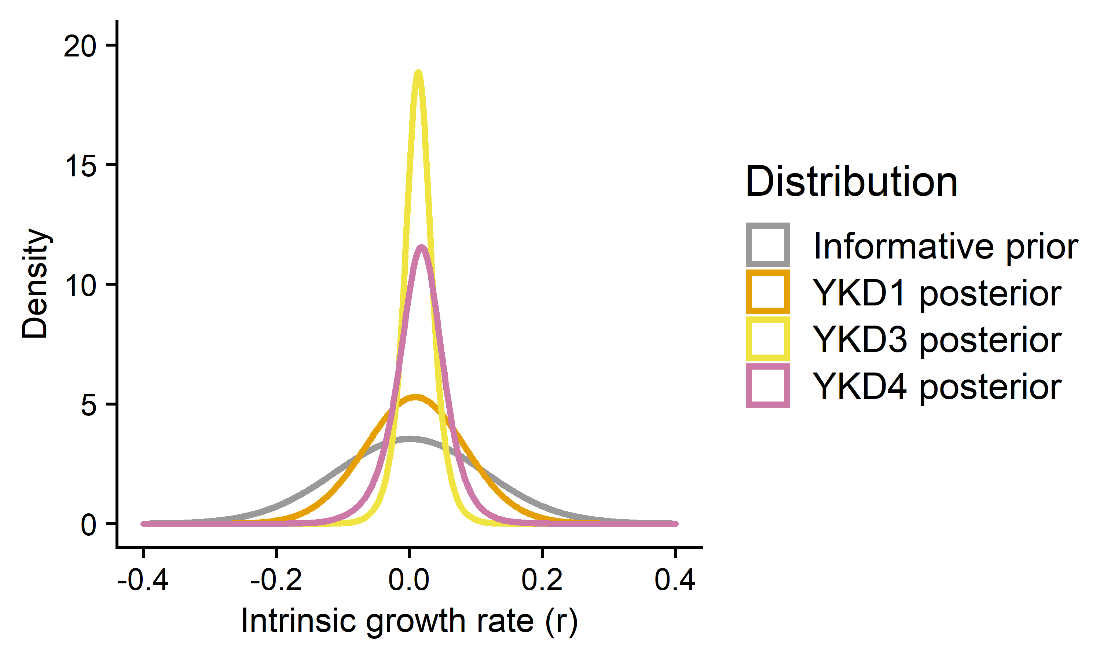


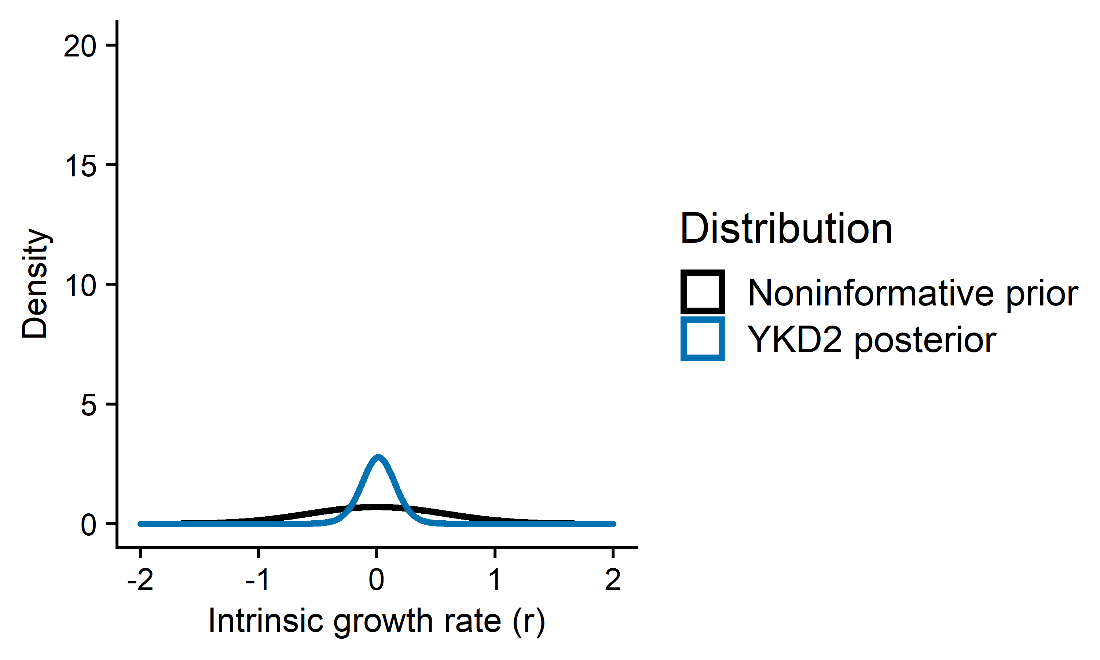


Fig S3. Prior distributions with the respective models’ posterior distribution for intrinsic population growth rate for spectacled eiders breeding on the Yukon Kuskokwim Delta. A weakly informative prior distribution was used to initialize models YKD1, YKD3, and YKD4 and reflects biologically plausible values elicited from expert opinion and species biology (top figure). A noninformative (diffuse) prior distribution was used to initialized model YKD 2 to reflect uncertainty in the possible values of population growth rates (bottom figure). Models YKD1, YKD3 and YKD4 were initialized with the same informative prior, however, the differed in the treatment of the observation process which resulted in similar mean estimates of population growth rate *r* but different distribution of possible values within the posteriors. Initializing model YKD2 with noninformative priors resulted in greater uncertainty in the posterior distribution compared to posteriors from models initialized with informative priors.


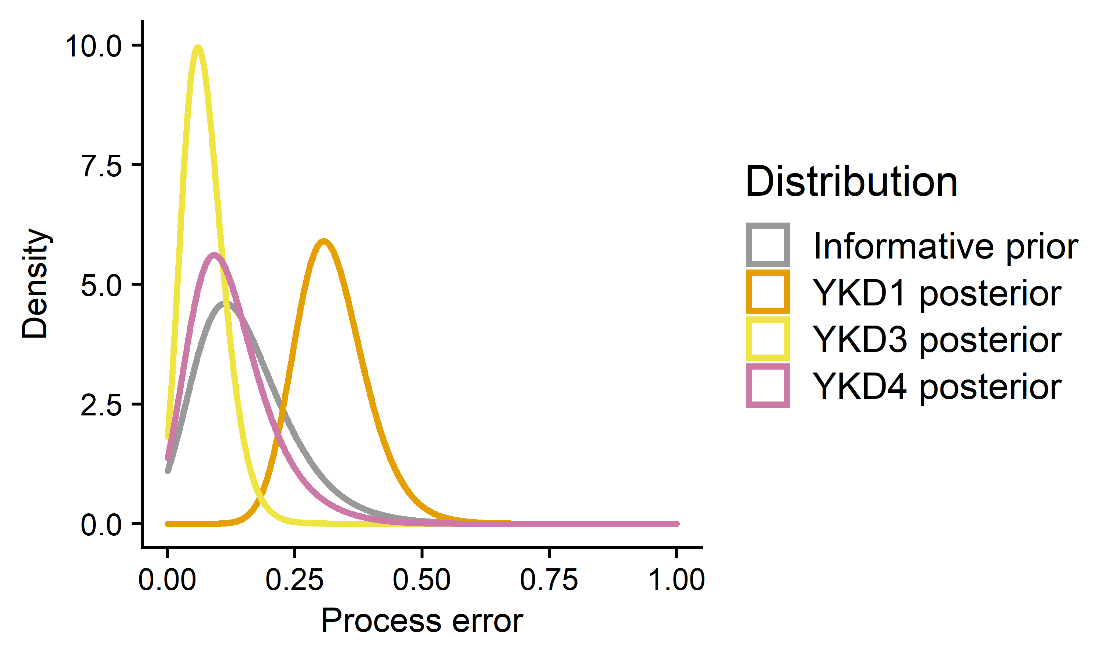


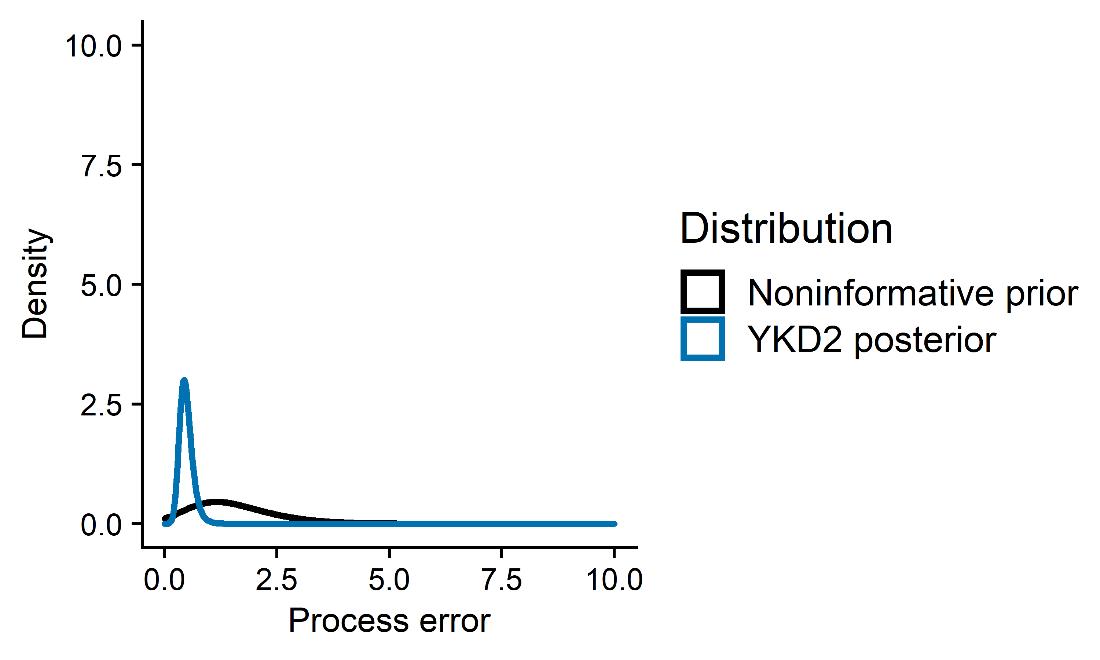


Fig S4. Prior distributions with the respective models’ posterior distribution for process variance (e.g., process error) for spectacled eiders breeding on the Yukon Kuskokwim Delta. A weakly informative prior distribution was used to initialize models YKD1, YKD3, and YKD4 and reflects biologically plausible values elicited from expert opinion and species biology (top figure). A noninformative (diffuse) prior distribution was used to initialized model YKD 2 to reflect uncertainty in the possible values of process error (bottom figure). Models YKD1, YKD3 and YKD4 were initialized with the same informative prior, however, the differed in the treatment of the observation process which resulted in differences in the mean and distribution of the posterior estimates. Initializing model YKD2 with noninformative priors resulted in greater uncertainty in the posterior distribution compared to posteriors from models initialized with informative priors.


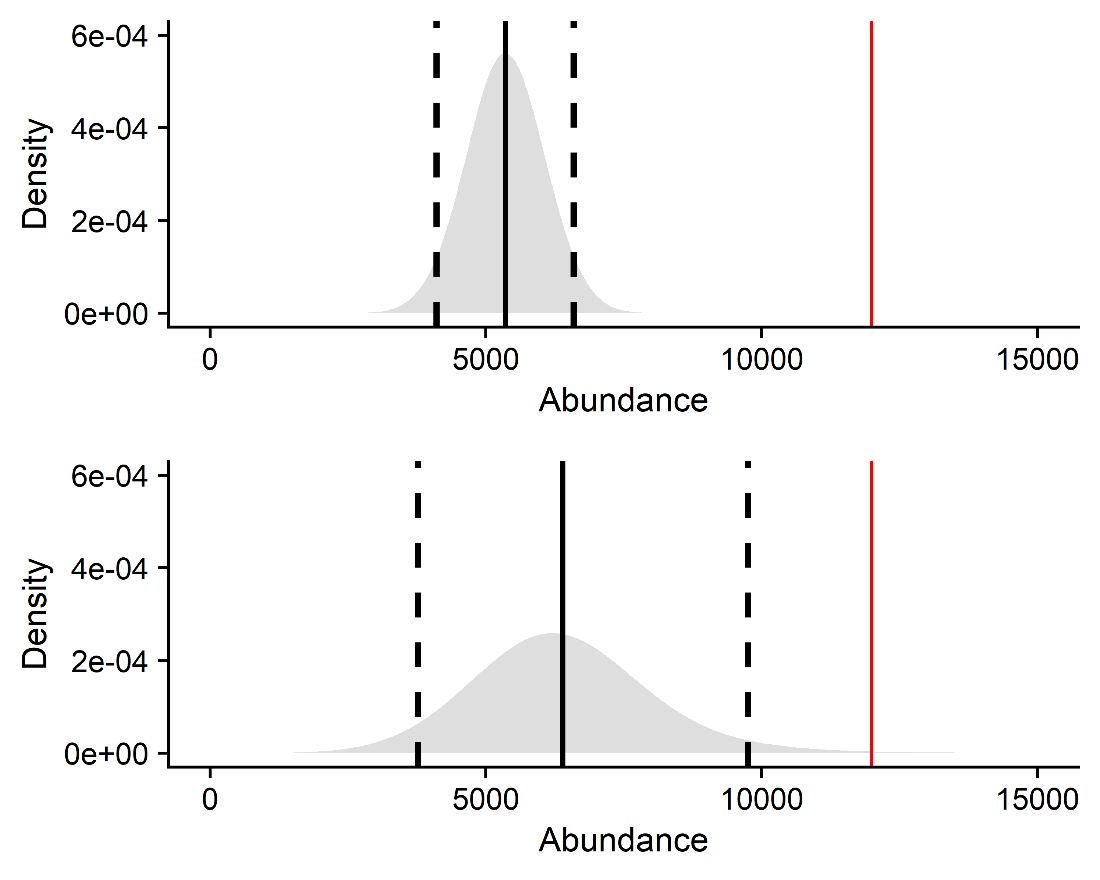


**ACP1**

**ACP2**

Fig S5. Posterior distributions of abundance in 2019 for spectacled eiders breeding on the Arctic Coastal Plain from two alternative models. The solid black vertical line indicates the mean, and the dashed black vertical lines represent the lower and upper 95% credible intervals. The solid red line is the abundance threshold set in the recovery plan as part of the measurable criteria for considering a decision to delist the species. To meet this criterion, the minimum estimated breeding population size must be $\geq$ 12,000 breeding birds designated by the 95% lower credible interval.


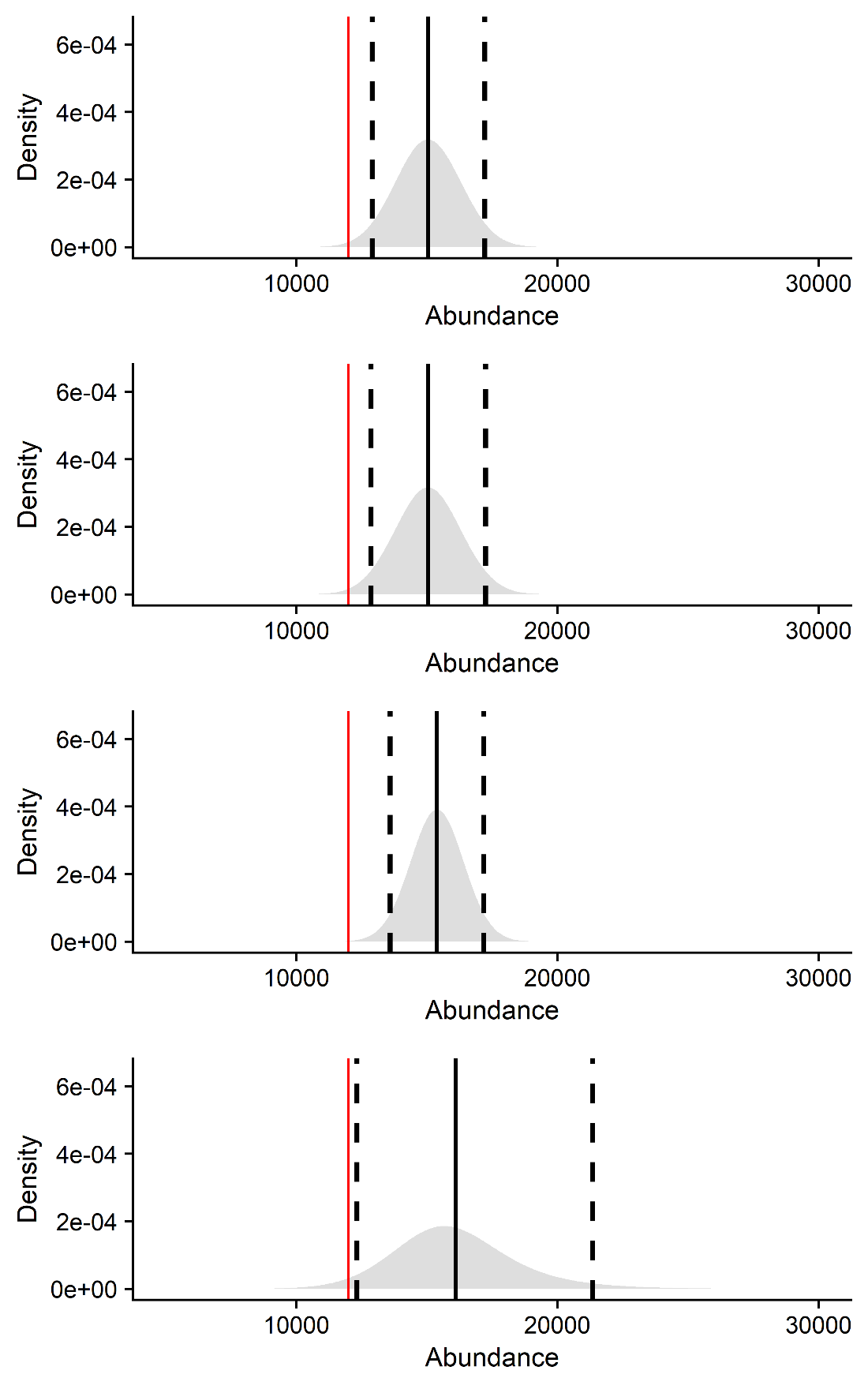


**YKD4**

**YKD3**

**YKD2**

**YKD1**

Fig S6. Posterior distributions of abundance in 2019 for spectacled eiders breeding on the Yukon Kuskokwim Delta from four alternative models. The solid black vertical line indicates the mean, and the dashed black vertical lines represent the lower and upper 95% credible intervals. The solid red line is the abundance threshold set in the recovery plan as part of the measurable criteria for considering a decision to delist the species. To meet this criterion the minimum estimated breeding population size must be $\geq$ 12,000 breeding birds designated by the 95% lower credible interval.
